# Characteristics and Consequences of Intimate and Non-Intimate Partner Stalking in Lithuania

**DOI:** 10.1177/08862605251336364

**Published:** 2025-05-03

**Authors:** Liubovė Jarutienė, Ilona Laurinaitytė, Ilona Michailovič, William J. Burk

**Affiliations:** 1 Vilnius University, Lithuania; 2 Radboud University, Nijmegen, The Netherlands

**Keywords:** intimate partner violence, stalking, harassment, coping strategies, victims

## Abstract

It is widely acknowledged that intimate partner violence can occur in various disturbing forms, including stalking behaviors. However, until recently, intimate partner stalking remained an understudied phenomenon in Lithuania. This study investigated the characteristics of intimate partner (IP) stalking, victims’ emotional reactions, and their coping strategies in a Lithuanian sample. A representative sample of 1,517 Lithuanian adults (*M*_age_ = 47.59 years, *SD* = 16.42) responded to an online survey. Of these, a total of 265 (18%) reported being stalked at least once during their lifetime, of which 102 reported being stalked by their current or former IP, of which 21 reported the IP stalking included physical violence. In contrast to victims of non-IP stalkers, victims of IP stalkers indicated that the perpetrators were more likely to make suicide threats, check the victim’s text messages via electronic devices, act aggressively upon seeing the victim out with others, and engage in both physical and sexual violence. Compared to victims of non-IP and nonviolent IP stalkers, victims of violent IP stalkers reported higher levels of anxiety, depression, fear, and helplessness. However, there were no significant differences between the victims of violent IP stalking, nonviolent IP stalking, and non-IP stalking in their coping strategies; moving away from the stalker was reported to be the most common coping strategy regardless of the victim type. The results of this study provide valuable insights about the characteristics of IP and non-IP stalking in Lithuania and reveal the consequences the stalking phenomenon might have on the victims’ physical and mental health.

## Introduction

Intimate partner (IP) stalking refers to various forms of repetitive unwanted behavior (e.g., showing up uninvited at one’s workplace and sending unwanted or threatening messages) perpetrated against current or former IPs that cause the victim to fear for their safety. IP stalking represents roughly 25% to 40% of stalking cases in the United States and European countries (Dressing et al., 2020; Laurinaitytė et al., 2022; Matos et al., 2019; Nobles et al., 2018). More importantly, the distinction between IP stalking and other types of stalking is important due to higher rates of recidivism (Eke et al., 2011) and a greater risk of physical violence among IP stalkers (Burczycka & Conroy, 2018; Logan, 2020; McEwan et al., 2017; Paterline, 2020). Thus, a substantial number of the most severe stalking cases occur in the context of an intimate relationship. In Lithuania, anti-stalking legislation has only been introduced recently (October 2021). However, for criminal liability to arise, the victim must suffer serious negative consequences, such as a change in residence, work, or educational institution, or the stalking must cause some other negative impact on his or her social life or emotional state (Laurinaitytė et al., 2022). Therefore, this may be considered as a questionable regulation as it requires victims to experience subjective fear or other similar emotional state, which may be difficult to prove. It is worth mentioning that, according to the most recent regulation in Lithuania, stalking is classified as a misdemeanor and a less serious criminal act making it impossible to apply a pretrial detention as a coercive measure. Moreover, the new anti-stalking regulation does not constitute a significant difference with respect to the focal comparison (IP vs. non-IP stalking), even though this distinction is substantial in Lithuanian scientific discourse on gender-based violence. Thus, it is important to better understand the prevalence, characteristics, and consequences of stalking, and especially IP stalking, to inform newly devised policies intended to support and protect victims. More importantly, since previous studies show that cultural context is associated with the prevalence as well as the nature of stalking (Chan & Sheridan, 2017; Sheridan et al., 2016, 2017) it is also important to explore the characteristics of stalking and, specifically, IP stalking in Lithuania since this country has experienced the periods of post-Soviet transition and rapid European integration and is still in search of adequate and effective response to various forms of IP violence.

## Characteristics of IP Stalkers and Their Victims

Similar to non-IP stalking, IP stalking can include a wide range of behaviors, but studies show that IP stalkers may exhibit some specific forms of stalking behavior more often. Compared to other stalkers, IP stalkers have been found to be more likely to make death threats and to seek unwanted contact with the victim through phone calls, excessive emails, text messages, and social media (Burczycka & Conroy, 2018). Compared to non-IP stalking, IP stalking is also more likely to involve behaviors such as showing up at the places where the victim can be met (Chan & Sheridan, 2022), trying to establish contact with others (Katz et al., 2020; White et al., 2022), and various surveillance tactics (Johnson & Thompson, 2016). IP stalking has also been reported to last longer, compared to other types of stalking (Burczycka & Conroy, 2018; Johnson & Thompson, 2016). In terms of victims’ characteristics, several studies have shown females and young people experience more stalking, and especially more IP stalking, than males and older adults (Augustyn et al., 2020; Burczycka & Conroy, 2018). Research on victims’ responses has shown that victims of IP stalking were more likely to report their experience to police, compared to victims of non-IP stalking (Burczycka & Conroy, 2018). These studies collectively indicate that victims experience the stalking behaviors of IPs to be especially problematic.

IP stalking has also been associated with a greater risk of physical violence compared to non-IP stalking (Burczycka & Conroy, 2018; Logan, 2020; McEwan et al., 2017; Paterline, 2020). The most recent studies show that women who were stalked by their ex-partners were twice as likely to be physically assaulted compared to women stalked by non-partners (Logan, 2024). In fact, roughly 35% to 40% of IP stalkers assault their victims at some point (Logan, 2024; McEwan et al., 2009; Vinas-Racionero et al., 2017). Prior studies also show that, among the victims of partner abuse, stalking was associated with a greater risk of life-threatening violence (Brady & Hayes, 2018). In general, IP stalking includes various forms of violence ranging from vandalism and destruction of the victim’s property (Burczycka & Conroy, 2018; White et al., 2022) to physical and sexual assault (White et al., 2022). Therefore, compared to non-IP stalkers, IP stalkers are more likely to engage in various acts of violence against their victims. Previous research indicates that victims of IP stalkers are at a greater risk of assault and, thus, IP stalking is associated with a variety of negative and harmful consequences for the victims who might be especially vulnerable due to their current or former intimate relationship with the stalker.

## Consequences of IP Stalking: Emotional Reactions and Coping Strategies

The consequences of stalking include negative emotional responses to the situation, as well as cognitive and behavioral strategies used by victims to cope with the stalker’s unwanted behavior. In fact, stalking victimization has been proven to have a negative impact on victim’s mental health (Matos et al., 2019; Short et al., 2015). According to the results of previous studies, victims of stalking often report experiencing fear, anxiety, anger, helplessness, and depressive symptoms as a result of stalking victimization (Acquadro Maran & Varetto, 2018; Borges & Dell’Aglio, 2019; Ngo & Paternoster, 2016; Nikupeteri, 2017; Podana & Imriškova, 2016; Worsley et al., 2017). Little is known about the impact of physical assault on the victim’s emotional response to stalking: some studies show that physical assault is related to higher levels of fear (Logan & Walker, 2021; Podana & Imriškova, 2016), however, the association between IP stalking violence and victim’s emotional reactions remains understudied.

It is important to explore the psychological consequences of the specific type of stalking and violent stalking,^1^ in particular, since some studies have revealed that the nature of stalking can result in specific emotional consequences for the victim (Villacampa & Pujols, 2019). Other studies, on the other hand, show that a victim’s emotional response to a stalking experience is often important from a legal point of view (Van der Aa, 2018). This is relevant for the new Lithuanian anti-stalking legislation, which includes a requirement for the victim to feel subjective fear or other negative emotions to start criminal proceedings. The studies on victims’ emotional response to violent IP stalking could inform further development of anti-stalking legislation in Lithuania and offer some options for proper legal response to violent stalking in the context of intimate relationships. Such studies might also encourage legislators to reconsider whether the legal criterion of negative emotional response is indeed universal and applicable to all stalking episodes. Finally, it is important to explore victims’ emotional response to violent IP stalking since it can affect victims’ choice of coping strategies (Ngo & Paternoster, 2016), which might determine the rates of reporting stalking to police and other types of victims’ help-seeking behaviors.

In addition to negative emotions, victims are also forced to utilize coping strategies to deal with the stalking experience (Ngo & Paternoster, 2016). Cupach and Spitzberg (2004) distinguish five types of coping strategies used by the victim of stalking: (a) *moving inward*—focusing primarily on oneself and one’s resources without any attempt to involve other people; (b) *moving outward*—relying on other people for their assistance and support; (c) *moving away*—avoiding the stalker and trying to limit one’s own accessibility; (d) *moving toward*—communicating, negotiating, and bargaining with the stalker to cease the pursuit; (e) *moving against*—actively resisting, retaliating, or threatening the stalker. Empirical studies have shown that moving away is the most common strategy among those who experienced stalking (Amar & Alexy, 2010; Chan & Sheridan, 2020). Some previous findings show that active coping strategies (e.g., seeking social or professional support, contacting law enforcement agencies, and taking actions to increase one’s safety) might be more advantageous for victim’s mental health (Kamphuis et al., 2003) and more effective in terms of stopping the stalking behavior (Dutton & Winstead, 2011; Geistman et al., 2013). Passive coping strategies such as withdrawal, avoidance, and rumination were found to increase the risk of posttraumatic stress among the victims of IP stalking (Kamphuis et al., 2003). There have not been many studies focusing on the coping strategies used by the victims of IP stalking, yet a few of these have shown that those victims tend to move away or outward to deal with the unwanted pursuit (Dutton & Winstead, 2011). While victims of stalking tend to “move away” from their stalkers, the prevalence of these coping strategies in victims of violent and nonviolent IP stalking has yet to be explicitly examined.

## Current Study

The main purpose of this study is to investigate differences between victims of IP and non-IP stalking in a representative sample of Lithuanian adults. Specifically, three research questions are addressed. First, do IP and non-IP stalking differ in characteristics of the victim, perpetrator, and stalking behavior? We expect victims of IP stalkers to be younger and more likely to report stalking to police compared to victims of non-IP stalking. Also, IP stalkers are expected to be more likely to make threats, initiate excessive unwanted communication through various platforms, damage victims’ property, apply various surveillance tactics, and be both physically and sexually violent compared to non-IP stalkers. Second, do victims of violent IP stalking, nonviolent IP stalking, and non-IP stalking differ in the emotions resulting from their stalking experience?^2^ We anticipate that victims of violent IP stalking will report more negative emotions (anxiety, depression, fear, and helplessness) compared to victims of nonviolent IP stalking and non-IP stalking. Third, do victims of violent IP stalking, nonviolent IP stalking, and non-IP stalking differ in the coping strategies they use in response to their stalking experience? We expect victims of violent IP stalking to report more moving away compared to victims of nonviolent IP and non-IP stalking, while victims of nonviolent IP stalking are expected to report more moving toward compared to victims of non-IP stalking.

## Method

### Participants

The initial sample consisted of 1,517 Lithuanian residents (54% female, *n* = 817) ranging in age from 18 to 87 years (*M* = 47.59 years, *SD* = 16.42). The sample was intended to be representative^3^ of the Lithuanian population and included the majority of adults who identified themselves as Lithuanian (84%, *n* = 1,280), other study participants represented ethnic minorities: 6% were Polish (*n* = 96), 5% were Russian (*n* = 79), and 4% (*n* = 62) represented other minorities such as Roma, Belarusian, and Ukrainian. A total of 57% were married (*n* = 857), 25% were single (*n* = 385), 9% were divorced (*n* = 137), and 9% specified their marital status as “other” (*n* = 135). In terms of employment status, 61% were currently employed (*n* = 922), 21% were retired (*n* = 313), 9% were unemployed (*n* = 137), and 9% (*n* = 143) reported another employment status.

The analytic sample consisted of 265 adults (17.5% of the initial sample) who reported being a victim of stalking. Participants were mostly female (63%, *n* = 168), and ranged from 18 to 77 years of age (*M* = 42.02 years, *SD* = 14.51). Most participants in this subsample (86%, *n* = 228) identified themselves as Lithuanian. A total of 48% were married (*n* = 127), 34% were single (*n* = 91), 15% were divorced (*n* = 39), and 3% (*n* = 7) specified their marital status as “other.” A total of 72% (*n* = 190) in this subsample were currently employed, 8% (*n* = 22) were retired, 11% were unemployed (*n* = 28), and 9% (*n* = 15) reported another employment status.

The non-victim sample consisted of 1,261 adults (82.5% of the initial sample). The majority of this sample were female (52%, *n* = 649), and ranged from 18 to 87 years of age (*M* = 48.77, *SD* = 16.56). Most participants in this sample identified themselves as Lithuanian (84%, *n* = 1,052), others identified themselves as the representatives of ethnic minorities, such as Polish (6.5%, *n* = 82), Russian (5.4%, *n* = 67), or other (e.g., Roma, Belarusian, Ukrainian, etc., 4.1%, *n* = 51). 23.5% of this sample were single (*n* = 294), 58.4% were married (*n* = 730), 7.8% claimed to be divorced (*n* = 98), and 10.3% reported other marital status (*n* = 128). During the study, 58.6% of participants (*n* = 782) were officially employed, 23.3% (*n* = 291) were retired, 8.7% (*n* = 109) were unemployed, and 9.4% (*n* = 118) reported another employment status. The comparison of those study participants who experienced stalking at least once during their lifetime and those who have never experienced stalking showed that the victims of stalking were significantly younger compared to non-victims (*t*(*df* = 422.95) = −6.71, *p* < .001). Also, compared to non-victims, victims’ included a higher percentage of females (63% vs. 52%, χ^2^(*df* = 2) = 11.89, *p* = .003), more divorced and single participants (15% vs. 7.8%, 34% vs. 23.5%, χ^2^(*df* = 4) = 40.03, *p* < .001) as well as more employed participants (72% vs. 58.6%, χ^2^(*df* = 5) = 32.32, *p* < .001) and a smaller percentage of retired participants (8% vs. 23.3%, χ^2^(*df* = 5) = 32.32, *p* < .001).

### Procedure

Study participants were initially contacted by the public opinion research company “Spinter Research.” Each participant was provided with detailed information about the study and only those who read and signed the informed consent form were further directed to the research questionnaire and completed the online survey. Considering the delicate and sensitive nature of the questions involving stalking experiences, participants who reported being a victim of stalking were encouraged to contact the study authors for psychological or legal assistance. Furthermore, all participants were provided with contact information for emotional support hotlines. The study was approved by the Ethics Committee for Psychological Research of Vilnius University (2021-04-13 No. 62), and the participants did not receive any financial or other kind of compensation for participating in the study.

### Measures

All participants completed questions that included demographic characteristics (e.g., gender, age, marital status). While collecting demographic data, race was not included as a separate characteristic since most residents in Lithuania represent the White/Caucasian racial group, while the share of the representatives of other racial groups is merely 0.2%. The participants were then provided with a definition of stalking and asked if they had experienced stalking at least once in their lifetime. In this study, stalking was defined as an intentional pattern of repeated behaviors (e.g., surveillance, life invasion, and property damage) toward a person or persons that are unwanted, resulting in fear, and can appear directly or via electronic means. Participants in the analytic sample (those who indicated that they had been stalked) also answered additional questions about the characteristics and behaviors of the stalkers, as well as their emotional reactions resulting from the stalking experience, and the coping strategies they used. Those participants who reported several distinct stalking experiences were asked to report about the most intense experience.

#### Characteristics of the Stalking Experience

Participants in the analytic sample received a list of 31 examples of stalking behavior and were asked to indicate those they had personally experienced. This list was based on previous research (Matos et al., 2019; McEwan et al., 2020; Nobles et al., 2018; Sheridan et al., 2001) and on the multidimensional framework of stalking assessment (Logan & Walker, 2017). Stalking behaviors were divided into four categories (Logan & Walker, 2017): *surveillance* (e.g., “Spying on you,” “Using mobile technologies to track your location”); *life invasion* (e.g., “Sending you unwanted letters, emails, notes, messages, or other written communication,” “Secretly using your computer, mobile phone, or electronic accounts,” “Denying that the relationship with you is over”); *intimidation* (e.g., “Damaging your property,” “Making threats of suicide or self-harm if you refuse to go out with them,” “Making threats to kill you or cause you physical injury”); *interference* through sabotage or attack (e.g., “Trying to harm your reputation,” “Being physically violent towards you (punching, kicking you, etc.),” “Trying to make you have sex with them”).

Participants also completed several items about the specific characteristics of the stalking experience: *duration* (1 month or less, from 1 to 6 months, from 6 to 12 months, from 1 to 2 years, more than 2 years), *frequency* (daily, weekly, monthly, less than once a month), *relationship with a stalker* (former or current IP vs. other^4^), and the stalker’s gender (male, female, and unknown).

#### Emotional Reactions to Stalking

Participants in the analytic sample completed items describing six emotions that might have been caused by the reported stalking experience (e.g., fear, anxiety, irritation, anger, depression, and helplessness). The response scale for these items consisted of a 5-point Likert scale, ranging from 1 (“Not at all”) to 5 (“Very much”).

#### Victims’ Coping Strategies

Participants also completed 26 items describing the five strategies proposed by Cupach and Spitzberg (2004): *moving inward* (e.g., “Minimized the problem in my own mind,” “Ignored the problem”); *moving outward* (e.g., “Sought sympathy from others,” “Sought help from others”); *moving away* (e.g., “Attempted to end the relationship,” “Restricted my own accessibility”); *moving toward* (e.g., “Attempted to deceive the stalker,” “Negotiated with the stalker”); *moving against* (e.g., “Issued verbal warnings,” “Used physical violence”). The response scale for these items consisted of a 5-point Likert scale, ranging from 1 (“Not at all”) to 5 (“Very much”). Each of these strategies demonstrated adequate internal reliability in this study: Cronbach alpha for *moving inward* scale was .61, for *moving away* scale—.78, for *moving toward* scale—.72, for *moving outward* scale—.81, for *moving against* scale—.72.

### Statistical Data Analysis

Independent samples *t*-tests (or chi-square analyses for categorical variables) were used to address the first research question involving differences between victims of IP and non-IP stalking. One-way ANOVAs were used to address the remaining research questions. In each ANOVA the independent variable was victim groups (violent IP, nonviolent IP, and non-IP stalking), and one of the measures of emotional reactions or coping strategies was the dependent variable. Pairwise comparisons with Bonferroni adjustment were used to test specific group differences following statistically significant omnibus *F*-tests. Analyses were performed using the IBM SPSS 27.0 (IBM Corp., 2020).

## Results

### Characteristics of Victims, Perpetrators, and Stalking Experience

Table 1 presents the differences between victims of IP and non-IP stalking on demographic characteristics of the victim, perpetrator, and the stalking experience. The results did not reveal any statistically significant age differences between victims of IP and non-IP stalking. No significant gender differences were found since most victims of both IP and non-IP stalking were female (64.7% vs. 62.6%). However, victims of IP stalking were less likely to be married and more likely to be divorced than victims of non-IP stalking. Considering the characteristics of the perpetrators, victims of IP stalking reported a higher proportion of female perpetrators compared to victims of non-IP stalking (32%, *n* = 33 vs. 21%, *n* = 34). The results of this study show that surveillance (Logan & Walker, 2017) was the most common stalking strategy, as the percentage of those who experienced at least one surveillance behavior was 89% (*n* = 91) in the IP stalking subsample and 80% (*n* = 131) non-IP stalking subsample. According to victims, overall, IP and non-IP stalkers did not differ in terms of the stalking strategies used. Focusing on specific stalking behaviors, victims of IP stalkers reported perpetrators were more likely to deny that the relationship was over (43%, *n* = 44 vs. 15%, *n* = 34), become aggressive upon seeing the victim with others (32%, *n* = 33 vs. 10%, *n* = 16), threaten to commit suicide (25%, *n* = 25 vs. 9%, *n* = 15), check the victim’s messages via electronic devices (28%, *n* = 28 vs. 12%, *n* = 19), be sexually violent (14%, *n* = 14 vs. 6%, *n* = 9), and assault the victim physically compared to non-IP stalkers (21%, *n* = 21 vs. 6%, *n* = 10).

**Table 1.** Victims’ Reported Characteristics of Intimate Partner and Non-Intimate Partner Stalking.

Characteristics of stalking cases	Non-IP stalking subsample (*n* = 163)		IP stalking subsample (*n* = 102)		*t*
	*M*	*SD*	*M*	*SD*	
Victim’s age	41.89	14.45	42.24	14.67	0.19
	*n*	%	*n*	%	χ^2^
Victim’s gender					
Male	61	37.4	36	35.3	.05
Female	102	62.6	66	64.7	
Victim’s ethnicity					
Lithuanian	143	87.7	85	83.3	.68
Other	20	12.3	17	16.7	
Victim’s marital status					
Single	53	32.7	38	37.2	30.62***
Married	95	58	33	32.4	
Divorced	10	6.2	29	28.4	
Other	5	3.1	2	2	
Victim’s occupational status					
Employed	112	68.7	78	76.5	1.50
Other	51	31.3	24	23.5	
Victim’s educational level					
Upper secondary	124	76.1	76	74.5	.02
Secondary or lower	39	23.9	26	25.5	
	*n*	%	*n*	%	χ^2^
Stalking duration					
1 month or less	36	22.1	27	26.4	3.58
1 to 6 months	55	33.7	31	30.4	
6 to 12 months	22	13.5	19	18.6	
1 to 2 years	18	11	12	11.8	
Longer than 2 years	32	19.6	13	12.7	
Stalking frequency					
Daily	49	30.1	29	28.4	3.27
Weekly	67	41.1	51	50	
Monthly	22	13.5	13	12.7	
Less than once a month	25	15.3	9	8.8	
Stalker’s gender					
Male	120	73.6	67	65.7	6.07
Female	34	20.9	33	32.3	
Mixed group or unknown	9	5.5	2	2	
	*n*	%	*n*	%	χ^2^
Disclosed victimization	63	38.7	40	39.2	.01
Reported stalking to police	22	13.5	15	14.7	.08
	*n*	%	*n*	%	χ^2^
Stalking behaviors					
Stalking strategy (Logan & Walker, 2017)					
Surveillance	***121***	***80.4***	***91***	***89.2***	***2.99***
Checking victim’s text messages via electronic devices	19	11.7	28	27.5	9.67**
Demanding victim’s electronic passwords	5	3.1	9	8.8	3.08
Secretly using victim’s computer/social media	12	7.4	14	13.7	2.2
Following	47	28.8	30	29.4	.01
Spying	58	35.6	38	37.3	.02
Using mobile technologies to check victim’s location	27	16.6	19	18.6	.07
Tracking victim’s activity on social media/internet	60	36.8	32	31.4	.6
Filming or taking pictures without permission	28	17.2	13	12.7	.63
Trying to obtain information about the victim from others	35	21.5	26	25.5	.37
Interference through sabotage or attack	***92***	***56.4***	***67***	***65.7***	***1.87***
Refusing to accept that a prior relationship with the victim is over	24	14.7	44	43.1	25.08***
Acting aggressively when seeing victim out with others	16	9.8	33	32.4	19.68***
Trying to make the victim have sex with them	9	5.5	14	13.7	4.34*
Physical violence	10	6.1	21	20.6	11.33***
Posting negative information about the victim	23	14.1	7	6.9	2.6
Impersonating the victim in emails, text messages, and/or social media	6	3.7	9	8.8	2.22
Insulting the victim	40	24.5	32	31.4	1.16
Threatening behavior toward victim’s family or friends	22	13.5	14	13.7	.001
Doing things to harm victim’s reputation	46	28.2	30	29.4	.005
Intimidation	***60***	***36.8***	***46***	***45.1***	***1.48***
Suicide threats	15	9.2	25	24.5	10.31***
Death threats	21	12.9	20	19.6	1.69
Damaging or vandalizing victim’s property	16	9.8	13	12.7	.29
Threatening victim by text, mail, or social media	30	18.4	16	15.7	.16
Life invasion	***102***	***62.6***	***71***	***69.6***	***1.08***
Communicating with the victim using other person’s account	7	4.3	10	9.8	2.32
Showing up uninvited	41	25.2	25	24.5	.01
Unwanted messages	48	29.4	30	29.4	.001
Unwanted phone calls	61	37.4	43	42.2	.41
Unwanted gifts	18	11.0	16	15.7	.83
Breaking into victim’s house	28	17.2	19	18.6	.02
Stealing or taking victim’s personal items	21	12.9	9	8.8	.67
Using text messages to harass the victim	29	17.8	21	20.6	.16
Giving victim’s personal information to others	32	19.6	16	15.7	.42

*Note*. IP = intimate partner.

* *p* < .05. **p < .01. ***p < .001

The two subsamples did not vary in terms of reporting stalking to the police, with only 14% (*n*_IP stalking subsample_ = 15 and, *n*_non-IP stalking subsample_ = 22) of victims reporting that they contacted the police. Thus, despite the higher rates of stalkers’ physical violence, victims of IP stalking were not more eager to report the stalking behaviors to law enforcement agencies.

### Emotional Reactions and Coping Strategies of Victims

The second aim of this study was to examine whether victims of violent IP stalking, nonviolent IP stalking, and non-IP stalking differ in terms of their emotional response to stalking. Table 2 presents the means and standard deviations of six negative emotions for the three victim types. The results indicate that the three victim types differed on four of the six emotions: anxiety, *F*(2, 262) = 3.84; *p* = .020; depression, *F*(2, 262) = 10.33, *p* < .001); fear, *F*(2, 262) = 6.75, *p* < .001); and helplessness, *F*(2, 262) = 8.75, *p* < .001. The three victim types did not differ in anger, *F*(2, 262) = 1.59, *p* = .206, or irritation, *F*(2, 262) = 1.89, *p* = .153. Pairwise comparisons (with Bonferroni adjustment) indicated that victims of violent IP stalking reported more anxiety, depression, fear, and helplessness than victims of nonviolent IP stalking and non-IP stalking. More importantly, victims of violent IP stalking reported the highest levels of anxiety, depression, and helplessness of all victim types.

**Table 2.** Emotional Responses and Coping Strategies of Victims of Non-Intimate Partner, Nonviolent Intimate Partner, and Violent Intimate Partner Stalking.

			IP (*n* = 102)			
	Non-IP (*n* = 163)		Nonviolent IP (*n* = 81)		Violent IP (*n* = 21)	
	*M*	*SD*	*M*	*SD*	*M*	*SD*
Emotional responses						
Anger	4.16	0.99	4.04	1.07	4.48	0.93
Anxiety	3.77_a_	1.18	3.77_a_	1.24	4.52_b_	1.12
Depression	3.26_a_	1.29	3.05_a_	1.37	4.48_b_	0.87
Fear	3.17_a_	1.44	3.00_a_	1.45	4.29_b_	1.45
Helplessness	3.66_b_	1.40	3.06_a_	1.41	4.48_c_	1.03
Irritation	4.23	0.94	4.25	1.04	4.67	0.91
Coping strategies						
Moving against	1.56	0.71	1.53	0.86	1.81	0.78
Moving away	3.27	0.94	3.22	0.85	3.69	0.84
Moving inward	2.88	0.90	2.91	0.79	2.44	0.78
Moving outward	2.17	0.84	2.14	0.80	2.01	0.70
Moving toward	2.17	0.92	2.38	0.87	2.51	0.92

*Note*. Within rows, means with different subscripts indicate statistically significant differences (*p* < .05) with Bonferroni adjustment. Across rows, different subscripts (a, b, and c) indicate statistically significant differences. IP = intimate partner.

The third aim of this study was to compare the coping strategies used by the victims of non-IP stalkers, nonviolent IP stalkers, and violent IP stalkers. The results revealed that the three victim types did not differ on any of the five coping strategies: moving against, *F*(2, 262) = 1.15, *p* = .317; moving away, *F*(2, 262) = 2.34, *p* = .098; moving inward, *F*(2, 262) = 2.65, *p* = .073; moving outward, *F*(2, 262) = 0.36, *p* = .698; moving toward, *F*(2, 262) = 2.38, *p* = .095. Although the mean-level differences between the three victim types were not statistically significant, victims of violent IP stalkers reported slightly higher levels of moving away, moving against, and moving toward the stalker than the other two victim types.

## Discussion

The main purpose of this study was to investigate the characteristics of IP stalking experiences from the victims’ perspective and to examine the consequences (emotional reactions and coping strategies) of non-IP stalking, violent, and nonviolent IP stalking in Lithuania. Generally, the results suggest that despite the fact that, in Lithuania, IP stalking has its own specific characteristics, victims of nonviolent IP and violent IP stalking do not differ in terms of the coping strategies used although stronger emotional reactions are reported in cases of violent IP stalking.

### Characteristics of Victims, Perpetrators, and Stalking Experience

The results of this study are partially in accordance with the hypothesis that, compared to non-IP stalkers, IP stalkers are more likely to make threats, use physical and sexual violence against their victims, engage in excessive unwanted communication, and damage the victim’s property. The findings suggest that, in Lithuania, IP stalkers were reported to be more likely to threaten to commit suicide, check the content of victim’s messages, deny that the relationship with the victim ended, act aggressively after seeing the victim with other people, and be sexually violent, compared to other types of stalkers. These tendencies are only partially in accordance with the results of previous research: other authors have also reported higher odds of sexual violence (White et al., 2022), death threats, and suicidal ideations among IP stalkers (Burczycka & Conroy, 2018; Katz et al., 2020; Racine & Billick, 2014). In contrast to other research, in Lithuania, IP stalkers did not differ from non-IP stalkers in terms of trying to contact the victim through a third party (Katz et al., 2020; White et al., 2022) nor in terms of damaging victim’s property (Burczycka & Conroy, 2018; White et al., 2022). The results of this study also replicate reports from other countries that indicate IP stalkers are predominantly male, and the victims of IP stalking are mostly young females (Augustyn et al., 2020; Burczycka & Conroy, 2018; Creamer & Hand, 2022). However, in Lithuania, victims’ reported rates of physical assault were lower compared to those reported in other countries (21% vs. 33%–38%; Logan, 2024; McEwan et al., 2009; Vinas-Racionero et al., 2017). Overall, the results of this study suggest that IP stalking behaviors are not entirely universal across various cultures, while some stalking behaviors are closely linked to the type of victim-stalker relationship. Although there could be some cultural differences, in many countries, including Lithuania, IP stalking is clearly associated with a greater risk of threats, coercive control, and sexual and physical violence. Therefore, correctional interventions for IP stalkers should target their attitudes toward IP violence as well as their interpersonal skills. Furthermore, the findings of this and previous studies should be reflected upon while developing a legal framework and considering effective response measures to ensure victims‘ safety. Considering specific risks posed by some IP stalkers, legal acts aimed at protecting the victims of IP violence should include specific algorithms for applying security measures to the victims of IP stalking (e.g., instant imposition of the emergency barring order, professional safety planning assistance).

In general, this study indicates that in Lithuania, the most prevalent stalking behaviors reported by victims could be attributed to surveillance strategies, however, IP stalkers are more likely to apply interference strategies which have been previously associated with IP stalking (Logan & Walker, 2017). Furthermore, in his study, self-identified victims of IP stalking report lower rates of stalker’s physically violent behavior, which is quite unexpected considering the high level of IP violence in the state which has risen from 47.941 reports in 2017 to 54.102 reports in 2023 (Jarutienė & Laurinaitytė, 2023). The differences in violence rates might be related to the development of digital technologies and the emergence of cyberstalking, which has been frequently observed in the context of intimate relationships (Cavezza & McEwan, 2014; Nuttman-Shwartz et al., 2023). Some elements of IP stalking might be gradually moving to cyberspace, thereby replacing the traditional forms of IP stalking, which could reduce the rates of stalking and physical violence.

Overall, the results indicate that, in Lithuania, IP stalkers were more likely to engage in interference through sabotage or attack, which has been previously distinguished as one of the most prevalent tactics among IP stalkers (Logan & Walker, 2017). More specifically, self-identified victims of stalking reported that IP stalkers engaged in acts of physical and sexual violence made suicide threats, attempted to check the content of the victim’s messages, and refused to accept that their relationship with the victim was over. As most victims in this study were stalked by their former partners, it could be assumed that, according to victims, IP stalking might have emerged as a destructive reaction to the break-up, while IP stalkers intended to gain control over their former IPs. These results might be related to the sensitivity of IP stalkers to rejection and their tendency to control their partners, as previous studies showed that IP stalking was associated with anxious attachment style (Creamer & Hand, 2022) and ex-partner’s coercive control (Ornstein & Rickne, 2013). Anxious attachment style was additionally predictive of aggressive behavior among stalkers (Dutton & Winstead, 2006) while this study shows higher rates of physical assault specifically among IP stalkers, therefore, the occurrence of violent episodes could also be associated with specific psychological characteristics of those who engage in unwanted pursuit of their current or former IPs.

Focusing specifically on victims of IP stalking, the rates of reporting IP stalking to police are rather low and comparable to those observed in non-IP stalking subsample, which is not in line neither with our hypothesis nor with the results of the previous research (Burczycka & Conroy, 2018). One possible explanation for these results might be that, in Lithuania, little has been done to inform and educate communities about stalking (Laurinaitytė & Michailovič, 2020), while those, who identify themselves as victims of stalking are more likely to minimize it (Laurinaitytė et al., 2022) and, therefore, stalking might be perceived as a petty crime. However, some authors note the rates of reporting stalking to police also being low in other countries (Augustyn et al., 2020; Fernández-Cruz, 2021) which suggests that stalking might not be given its due attention worldwide. Therefore, the results of this and the previous studies collectively indicate the need for awareness-raising initiatives to inform communities about stalking, ways to recognize it, and legal and social support resources available for the victims in each specific country. In addition, sharing good practice examples of successfully managing cases of stalking and, by doing so, supporting the notion that competent agencies are able to help the victims of stalking which could presumably increase the rates of reporting stalking to law enforcement agencies. In Lithuania, recent initiatives to address the issues of stalking include further amendments to the Act Against Domestic Violence, and the introduction of the emergency barring order for the victims of domestic violence, creating a more extensive victim assistance network. Therefore, it can be assumed that future studies could possibly reveal some changes in the rates of reporting both IP and non-IP stalking to police.

### Emotional Reactions and Coping Strategies of Victims

This study also investigated Lithuanian victims’ emotional responses to their stalking experience. As was expected, the findings indicated victims of violent IP stalkers reported higher levels of anxiety, depression, fear, and helplessness, compared to the victims of non-IP and nonviolent IP stalkers, which is in accordance with the results of the previous studies (Logan & Walker, 2021; Podana & Imriškova, 2016). Fear has previously been distinguished as one of the primary negative emotions caused by IP stalking (Logan & Walker, 2021; Podana & Imriškova, 2016), while stalking, in general, was found to have negative consequences for victim’s mental health (Matos et al., 2019; Short et al., 2015). However, this study provides additional evidence that IP stalking, and especially violent IP stalking, is associated with a wide range of negative emotional reactions. Stronger emotional responses to violent IP stalking might be a result of exposure to several types of IP violence (stalking and physical assault) which has been found to be associated with a higher risk of mental health issues (Ansara & Hindin, 2011; Jiwatram-Negron et al., 2022). Therefore, in cases of violent IP stalking, victims’ needs for mental health services should be prioritized and properly met by victim service professionals. Furthermore, the results of this study indicate that including the victim’s negative emotional response to stalking as one of the criteria for criminal liability to arise might not be reasonable since the nature and intensity of the victim’s emotional response to unwanted pursuit is not universal. Related anti-stalking legislation has also been criticized by other authors emphasizing the variety of victims’ emotional reactions to stalking (Van der Aa, 2018). Since the results of this study confirm this notion, in some countries, including Lithuania, certain revisions of anti-stalking legislation should be made excluding the requirement for stalking to cause any specific consequences to the victim.

This study also examined the differences in coping strategies used by the victims of non-IP, nonviolent IP, and violent IP stalking. Somewhat surprisingly and contrary to what was expected, we did not detect differences between victims of non-IP and IP (both violent and nonviolent) stalkers in the coping strategies used to deal with stalking experience. Generally, regardless of the type of stalking, all victims reported that moving away (i.e., attempting to distance oneself from the stalker, avoiding any unwanted contact, and applying various measures to limit their accessibility) was the most prevalent type of coping strategy. Furthermore, according to the victims, moving against (i.e., taking legal actions against the stalker, confronting, or warning the stalker) was the least common coping strategy used to deal with stalking experience. While the three victim types did not differ in their use of these coping strategies, these findings are in line with the tendencies uncovered by previous research (Amar & Alexy, 2010; Chan & Sheridan, 2020; Dutton & Winstead, 2011). However, Johansen and Tjørnhøj-Thomsen (2016) argue that victim-centered coping strategies, which imply that victims take the initiative to cease stalking, might have negative social consequences for the victims (i.e., retaliation), and the prevalence of such coping strategies might indicate the failure of law enforcement agencies to respond to stalking properly. Considering that victims in this study reported that moving away was the most common coping strategy, it might be assumed that this specific strategy might be common due to the lack of other means of coping which forces the victims to rely on their personal resources. In other words, in cases of stalking, victims might not be receiving enough legal and psychological assistance from the state and nongovernmental organizations due to the insufficient amount of public attention given to the problem of stalking.

Since the victims of violent IP stalking did not differ in terms of their coping strategies from the victims of nonviolent IP and non-IP stalking, the results of this study indicate that even acts of violence and aggression might not lead to seeking social support or reporting stalking to law enforcement agencies, although moving against was previously distinguished as an effective coping strategy in cases of violent stalking (Geistman et al., 2013). On the other hand, moving against the stalker might be not as effective in Lithuania due to very limited opportunities for the application of anti-stalking measures. In Lithuania, the emergency barring order has been only recently introduced (July 2023) and can only be imposed on the perpetrators of domestic violence, while the imposition of the obligation to live apart from the victim or refrain from approaching during the pretrial investigation is uncommon (Laurinaitytė et al., 2022). These legal and practical circumstances could be the reasons why victims of violent IP stalking often do not take action to confront the stalker or report stalking to the police.

### Limitations and Future Research

This study has certain limitations that should be addressed during future explorations of IP stalking and its consequences. First, the study is based on self-reported data, which are susceptible to recall biases of the study participants. The recent developments in anti-stalking legislation in Lithuania could possibly facilitate the collection of data on IP stalking, in that, more cases will be officially registered, and information can be officially collected from both victims and perpetrators. Second, this study did not consider gender differences between stalkers and victims. According to the victims, in this study, 32% of IP stalkers were female, while 35% of victims of IP stalking identified as male. Although both non-IP and IP stalking are often considered a male-to-female perpetrated type of crime (Dressing et al., 2020; Matos et al., 2019), future studies could explore the differences between male-to-female and female-to-male IP stalking, as well as explore the characteristics of stalking among the members of LGBTQIA+ community. Third, while we were able to compare IP stalking victims who did and did not experience physical violence, we could not do the same for non-IP stalking victims due to the low number of participants (*n* = 10). Future studies utilizing stratified sampling procedures that oversample non-IP stalking victims, especially those experiencing physical violence, would allow for further differentiation between the impact of the perpetrator (IP vs. non-IP) and severity (violent and nonviolent) of the stalking experience. Finally, although a large representative sample was used for this study, and a large number of stalking victims were identified, statistical comparisons between the three victim groups (non-IP stalking, violent, and nonviolent IP stalking) did not account for potential confounding variables or other third-variable explanations. As a result, statistically significant (and nonsignificant) differences between the stalking groups reported in this study should be interpreted with caution. This study represents an initial effort to better understand the differences in the experiences of non-IP and IP (both violent and nonviolent) stalking victims. Future studies could increase the strength of inference regarding differences between stalking victim groups by matching participants based on demographic and personal characteristics (e.g., propensity score matching) or performing more complex statistical analyses that minimize biases due to confounding variables.

## Conclusions

The results of this study suggest that, according to victims’ IP stalkers can be characterized as posing a greater risk of other forms of IP violence and are more likely to disregard victim’s personal boundaries. Furthermore, victims of violent IP stalking reported higher levels of negative emotional reactions, although victims’ coping strategies did not differ depending on the type of stalking experienced (non-IP, nonviolent IP, or violent IP). Therefore, despite the greater danger posed by IP stalkers and the potential harm of IP stalking victimization to victims’ mental health, the predominance of victim-centered coping strategies could signify the lack of a proper legal and institutional response to stalking. The findings of this study also indicate that the legal requirement for stalking to cause any specific consequences to the victim is questionable since the consequences may vary depending on the type of stalking experienced. Overall, it may be concluded that stalking is a criminal act with severe and varied consequences for victims, requiring professional and evidence-based assistance. We believe that our study contributes to a better understanding of stalking and its prevention across different cultures, as well as informing policies for appropriate regulatory changes in the field of stalking.

## Notes

1. In this study, violent stalking refers to the episodes of stalking which include physical assault.

2. Since the number of violent non-IP stalking cases in the study sample was very small (*n* = 10), this group was not included in the statistical analyses.

3. According to the data provided by the Official Statistics Portal (2021), the share of female residents in Lithuania was 53.4%, while Lithuanians comprised 85.1% of the population. Among those reseidents above 15 years of age, 48% were officially married, 13.6% were divorced, and 9% were widowed.

4. Other relationship options included family members and relatives, friends, co-workers, and acquaintances, someone met on the internet, strangers, and other.
